# Gene expression profile and pathogenicity of biofilm-forming *Prevotella intermedia *strain 17

**DOI:** 10.1186/1471-2180-9-11

**Published:** 2009-01-16

**Authors:** Takeshi Yamanaka, Tomoyo Furukawa, Chiho Matsumoto-Mashimo, Kazuyoshi Yamane, Chieko Sugimori, Takayuki Nambu, Naoki Mori, Hiroyuki Nishikawa, Clay B Walker, Kai-Poon Leung, Hisanori Fukushima

**Affiliations:** 1 Department of Bacteriology, Osaka Dental University, Osaka, Japan; 2 Department of Oral Biology, College of Dentistry, University of Florida, Gainesville, FL 32610-0424, USA; 3 Microbiology Branch, U.S. Army Dental and Trauma Research Detachment, Walter Reed Army Institute of Research, Great Lakes, IL 60088, USA

## Abstract

**Background:**

*Prevotella intermedia *(*P. intermedia*), a gram-negative, black-pigmented anaerobic rod, has been implicated in the development of chronic oral infection. *P. intermedia *strain 17 was isolated from a chronic periodontitis lesion in our laboratory and described as a viscous material producing strain. The stock cultures of this strain still maintain the ability to produce large amounts of viscous materials in the spent culture media and form biofilm-like structures. Chemical analyses of this viscous material showed that they were mainly composed of neutral sugars with mannose constituting 83% of the polysaccharides. To examine the biological effect of the extracellular viscous materials, we identified and obtained a naturally-occurring variant strain that lacked the ability to produce viscous materials *in vitro *from our stock culture collections of strain 17, designated as 17-2. We compared these two strains (strains 17 versus 17-2) in terms of their capacities to form biofilms and to induce abscess formation in mice as an indication of their pathogenicity. Further, gene expression profiles between these two strains in planktonic condition and gene expression patterns of strain 17 in solid and liquid cultures were also compared using microarray assays.

**Results:**

Strain 17 induced greater abscess formation in mice as compared to that of the variant. Strain 17, but not 17-2 showed an ability to interfere with the phagocytic activity of human neutrophils. Expression of several genes which including those for heat shock proteins (DnaJ, DnaK, ClpB, GroEL and GroES) were up-regulated two to four-fold with statistical significance in biofilm-forming strain 17 as compared to the variant strain 17-2. Strain 17 in solid culture condition exhibited more than eight-fold up-regulated expression levels of several genes which including those for levanase, extracytoplasmic function-subfamily sigma factor (σ^E^; putative) and polysialic acid transport protein (KpsD), as compared to those of strain 17 in liquid culture media.

**Conclusion:**

These results demonstrate that the capacity to form biofilm in *P. intermedia *contribute to their resistance against host innate defence responses.

## Background

*Prevotella intermedia*, a gram-negative, black-pigmented anaerobic rod, is frequently isolated from periodontal pockets of patients with chronic periodontitis [1], acute necrotizing ulcerative gingivitis [2], pregnancy gingivitis [3], and endodontic lesions [4-6]. This organism possesses a number of virulent factors that underlie it's pathogenic potential for causing infections [7-11].

*P. intermedia *strain 17 was initially isolated from a chronic periodontitis lesion in our laboratory [12] and some of its phenotypic characteristics were determined. Among these included the ability of the organism to: (a) produce viscous materials *in vitro *[12]; (b) invade human oral epithelial cells [13]; and (c) stimulate CD4^+ ^T cells expressing Vβ8, Vβ12 and Vβ17 [14]. More recently, the whole genome sequence of strain 17 was determined by The Institute for Genomic Research (TIGR; Rockville, MD, USA) [15].

In our earlier study, we demonstrated that a clinical isolate of *Prevotella nigrescens *is able to produce extracellular viscous material that might contribute to its biofilm formation [16]. In this context, we hypothesized that the ability of *P. intermedia *strain 17 to produce viscous materials might be essential for its biofilm formation. In this study, we describe the chemical composition of the viscous materials as determined by means of high performance liquid chromatography (HPLC) and colorimetry. To define the role of the extracellular viscous materials in biofilm formation, we identified and obtained a naturally-occurring variant strain that lacked the ability to produce viscous materials *in vitro *from our stock culture collections of strain 17, designated as 17-2. We compared the ability of these two strains (strains 17 versus 17-2) in their ability to form biofilms and to induce abscess formation in mice as an indication of their pathogenicity. Further, we sought to determine the gene expression profiles associated with the biofilm formation by these two strains using microarray assays.

## Results

### Viscosity of spent culture medium

**Figure 1:**
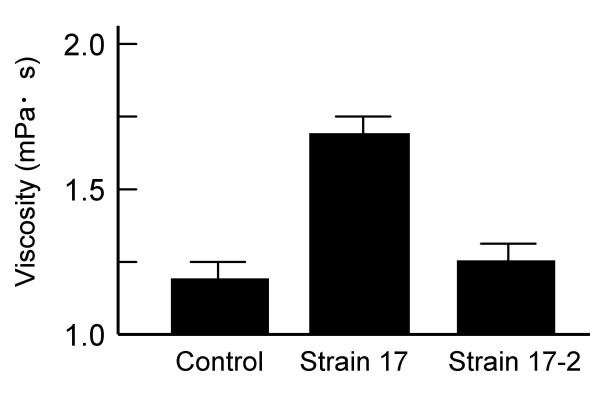
**Viscosities of the spent culture media of *Prevotella intermedia *strains 17 and 17-2**. Viscosities of the spent culture media obtained from *Prevotella intermedia *strains 17 and 17-2 were measured by a rotary viscometer. The viscosity of the enriched-TSB medium was measured as a control. Bars indicate standard deviations.

Stock cultures of *P. intermedia *strain 17 were transferred to enriched-trypticase soy broth (enriched-TSB) and grown for 48 h. The viscosities of spent culture media were measured by a rotary viscometer. All tested *P. intermedia *strain 17 stocks, with the exception of one particular stock strain, designated as strain 17-2, produced materials *in vitro *that were highly viscous as compared to the control TSB medium. In contrast, the viscosity of spent culture medium obtained from strain 17-2 was similar to that of the control TSB medium (Fig. 1).

### Cell surface associated structures

**Figure 2:**
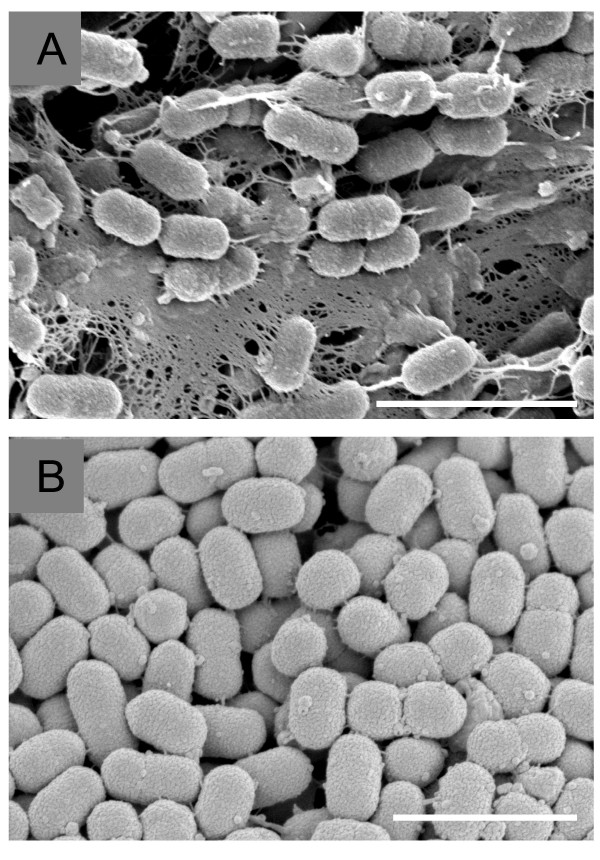
**Cell surface structures of *Prevotella intermedia *strains 17 and 17-2**. Scanning electron micrographs showing the surface structures of *Prevotella intermedia *strains 17 and 17-2. The specimen was prepared from a colony of each strain grown on a blood agar plate. Strain 17 had dense meshwork-like structures surrounding the cell surfaces (A), but strain 17-2 lacked this phenotype (B). Bars = 2 μm.

SEM observations on cells from colonies of these strains growing on blood agar plates revealed that strain 17 had dense meshwork-like structures around the cells (Fig. 2A), but strain 17-2 lacked this phenotype (Fig. 2B). The lack of abilities to produce viscous materials in culture medium and to form meshwork-like structures around cells on strain 17-2 were stably maintained despite repetitive passages *in vitro *or in animals (data not shown).

### Biofilm formation assay

**Figure 3:**
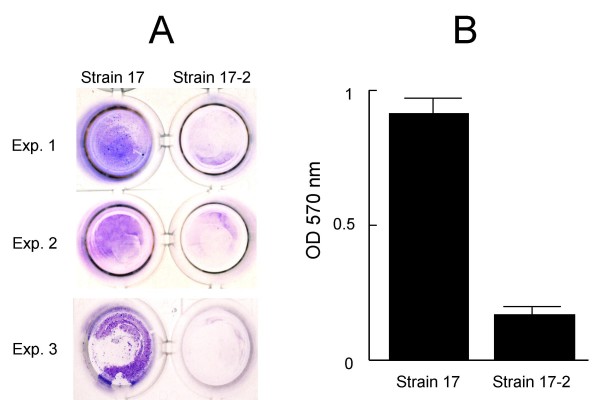
**Biofilm formation on microtiter plates**. Biofilm production of *Prevotella intermedia *strains 17 and 17-2 on polystyrene microtiter plates: a representative pair of microtitier plate wells from each experiment stained with 0.1% crystal violet solution after 24 h of incubation (A). The quantitative analysis of biofilm production as measuring the optical density of destained biofilms at 570 nm (B). Bars indicate standard deviations.

The ability to form biofilm was investigated for strains 17 and 17-2 using crystal violet microtiter plate assay. Strain 17 was consistently able to form biofilm on flat-bottomed polystyrene microtiter plates, whereas strain 17-2 showed poorer biofilm formation (Fig. 3A). Quantitative analysis as measuring the optical density of destained biofilms at 570 nm revealed that the ability of strain 17 to form biofilm was significantly greater than that of strain 17-2 (p < 0.01) (Fig. 3B).

### Morphology and chemical composition of the viscous materials

**Figure 4:**
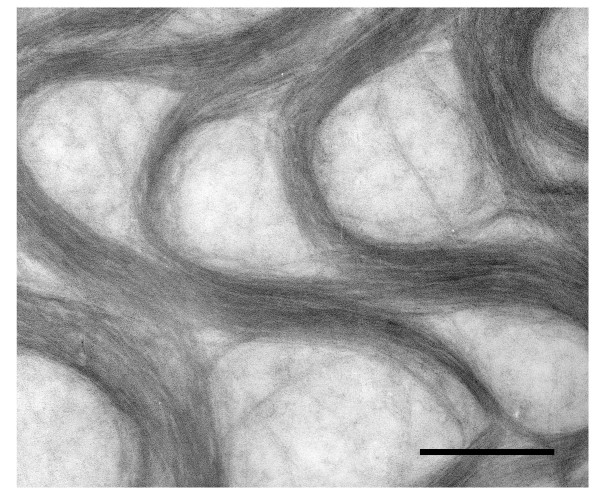
**Transmission electron microscopy of negatively stained exopolysaccharides isolated from *Prevotella intermedia *strains 17 culture supernatants**. Note the fine fibrous structures that are formed in bundles. Bar = 500 nm.

Negative staining of the viscous material isolated from strain 17 culture supernatants revealed that the viscous material was made up of fine fibrous structures formed in curly bundles (Fig. 4). Chemical analyses of this purified material showed that it primarily consisted of neutral sugars and small amounts of uronic acid and amino sugars (Table 1), with mannose constituting 83% of the polysaccharide (Table 2).

**Table 1:** Amount of neutral sugar, uronic acid and amino-sugar in the viscous material isolated from *Prevotella intermedia *strain 17

Sugar	Amount (μg/mg)
Neutral sugar	795.5
Uronic acid	28.8
Amino-sugar	11.3

**Table 2:** Neutral sugar components of the exopolysaccharide isolated from *Prevotella intermedia *strain 17

Neutral sugar	Amount (μg/mg)
Mannose	684.7
Glucose	53.5
Galactose	29.2
Arabinose	17.3
Xylose	5.8
Rhamnose	2.8
Ribose	2.2

### Gene expression profiles of *P. intermedia *strains 17 and 17-2

**Figure 5:**
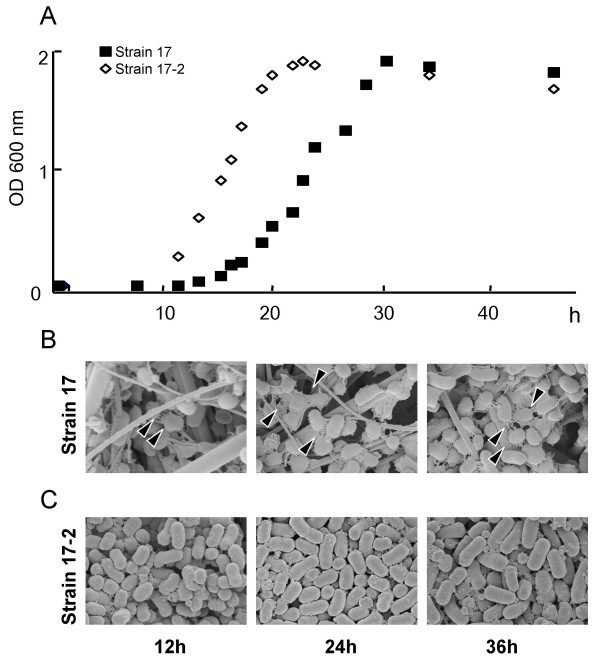
**Growth of *P. intermedia *strains 17 and 17-2 in enriched-trypticase soy broth and scanning electron micrographs showing morphological changes associated with growth**. Strains 17 and 17-2 entered into the exponential phase at a different time point. Strain 17-2 shows a faster growth rate (A). Meshwork-like structures around strain 17 cells were observed at 12 h and became denser with time. Arrowheads indicate cells with meshwork-like structures (B). No such morphological changes were observed in strain 17-2 (C).

To see what kind of gene expression events induce phenotypic differences on *P. intermedia*, we compared gene expression patterns between strains 17 and 17-2, the respective viscous material producing and non-producing strains using microarray analysis. To determine the appropriate time point for isolating total RNA, we first observed the morphological changes of cell surface structures in each strain along with the bacterial growth. In general, the growth of strain 17-2 was faster than that of strain 17, entering into an exponential phase at around 12 h and reaching the plateau in 24 h (Fig. 5A, open rhombus). Strain 17-2 did not show the presence of cell-associated fibrous materials at any stage of the growth cycle (Fig. 5C). By contrast, strain 17 showed a slower growth rate (Fig. 5A, hatched square) with a longer exponential growth phase. Morphological observation of cultures at different stages of growth revealed that strain 17 exhibited cell surface-associated meshwork-like structures at 12 h and the structures became denser with time (Fig 5B). From these preliminary data, 12 h-old cultures of strains 17 and 17-2 were chosen for a comparison of gene expression patterns. When the microarray expression data for strains 17 and 17-2 were compared, a total of 11 genes were up-regulated by at least two-fold with statistic significance (p < 0.05) in biofilm-forming *P. intermedia *strain 17 (Table 3). The expression data demonstrated that several heat shock protein (HSP) genes, such as *dnaJ, dnaK, groES, groEL and clpB *were up-regulated in strain 17 (Table 3). We also identified two genes down-regulated at least two-fold in strain 17 (PINA2115: hypothetical protein; PINA2117: sterol-regulatory element binding protein (SREBP) site 2 protease family). The original raw data files have been deposited in Center for Information Biology gene Expression database (CIBEX; Mishima, Japan; CIBEX accession: CBX27) [17].

**Table 3:** Genes showing at least two-fold higher expression levels in biofilm-forming *Prevotella intermedia *strain 17 than those of non-forming variant strain 17-2

Gene	Fold change	Annotation
PIN0258	2.63	Hypothetical protein
PIN0281	3.42	Heat shock protein 90, HtpG
PINA0419	2.17	Hypothetical protein
PINA0775	2.47	Patatin-like phospholipase family protein
PINA1058	2.28	DnaK protein
PINA1693	2.09	Folylpolyglutamate synthase, FolC
PINA1756	2.35	Heat shock protein, DnaJ
PINA1757	2.31	Hypothetical protein
PINA1797	2.33	Chaperonin, 60 kDa, GroEL
PINA1798	2.39	Chaperonin, 10 kDa, GroES
PINA2006	2.17	ClpB protein

### mRNA levels for HSPs validated by real-time RT-PCR

**Figure 6:**
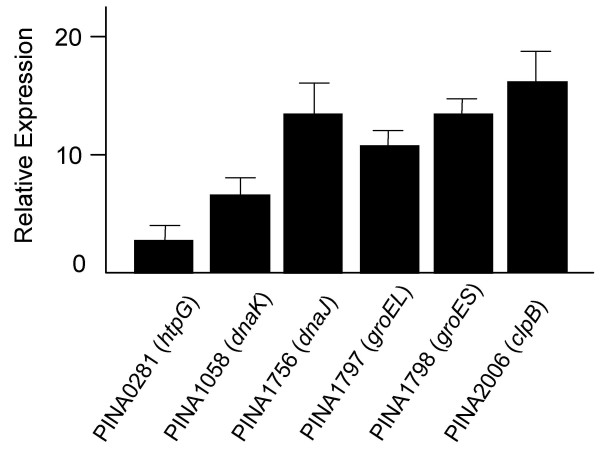
**Validation of the up-regulation of five heat shock protein genes (PINA1058, PINA1756, PINA1797, PINA1798, PINA2006) in strain 17 by quantitative real-time RT-PCR**. Total RNA was isolated from 12 h-old cultures of strains 17 and 17-2, and the expression levels of these genes were compared by real-time RT-PCR. The average of increased expression level of PIN 0281 was less than twofold in the real-time RT-PCR analysis though a three-fold up-regulation of this gene was observed by the microarray assay.

In the microarray analysis, we identified that several of the heat shock protein genes were up-regulated in strain 17 as compared with those of strain 17-2. The increased expression levels of these genes were validated in an independent experiment by real-time RT-PCR using the 16S rRNA gene as the endogenous control. Annotations of these genes (PIN0281, PINA1058, PINA1756, PINA1797, PINA1798, and PINA2006) on TIGR data base were described in Table 3. Except PIN0281, five out of six of tested genes showed an at least fivefold increased average expression levels in strain 17 as confirmed by the quantitative real-time RT-PCR. Although PIN0281 showed about a three-fold up-regulation in strain 17 by the microarray analysis, the average of increased expression level of PIN0281 was less than two-fold in the real-time RT-PCR analyses (Fig. 6).

**Figure 7:**
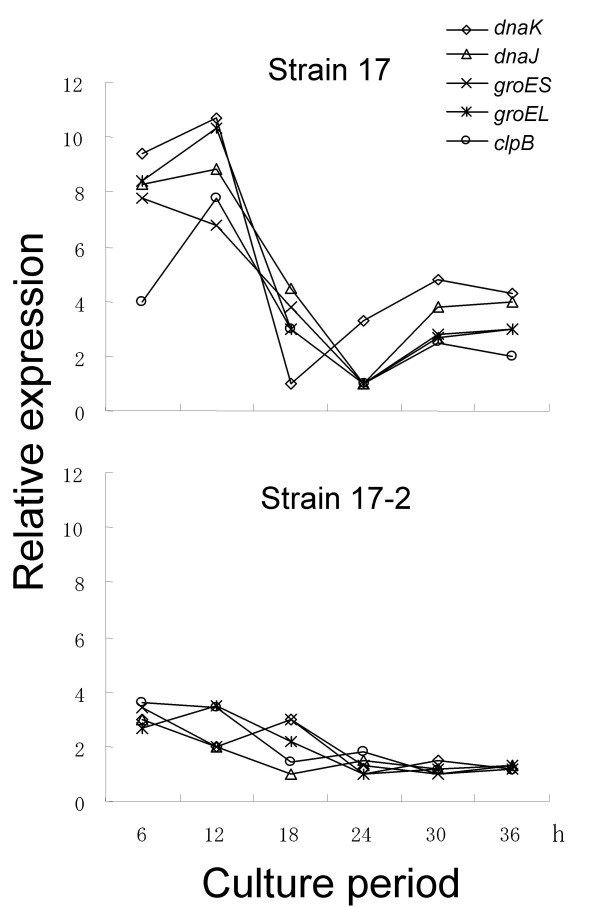
**Fluctuation of transcriptional levels of heat shock protein genes through a culture period in strains 17 and 17-2**. Total RNA was isolated from 6, 12, 18, 24, and 30 h-old cultures of strains 17 and 17-2, and the relative expression levels of these genes were recorded by the strain using real-time RT-PCR. The expression levels of these genes were fluctuating in strain 17 but not in strain 17-2. Data are representative of two independent experiments. *dnaK*: PINA1058; *dnaJ*: PINA1756; *groEL*: PINA1797; *groES*: PINA1798; *clpB*: PINA2006.

The data obtained from the microarray analysis as well as the real time RT-PCR showed that several of HSP genes were up-regulated in strain 17 in 12 h-old cultures as compared with those of strain 17-2. Next, we addressed the question of whether the different expression levels of HSP genes between the two strains are due to a lag of growth because strain 17 showed a slower growth rate than that of strain 17-2 (Fig. 5). The relative expression levels of HSP genes through the culture period were obtained using real time RT-PCR by the strain. In strain 17, the expression levels of these genes were fluctuating; increased in early exponential phase (6 h to 12 h), decreased once in the middle of exponential phase (18 h to 24 h), and then slightly increased again in early stationary phase. By contrast, strain 17-2 did not show such fluctuated transcriptional levels in all HSP genes through the culture period (Fig. 7). Judging from the comparison between strains 17 and 17-2 at 12 h-old cultures (Fig. 6), strain 17-2 seems to keep the expression levels of these HSP genes very low.

### Abscess induction in mice

**Figure 8:**
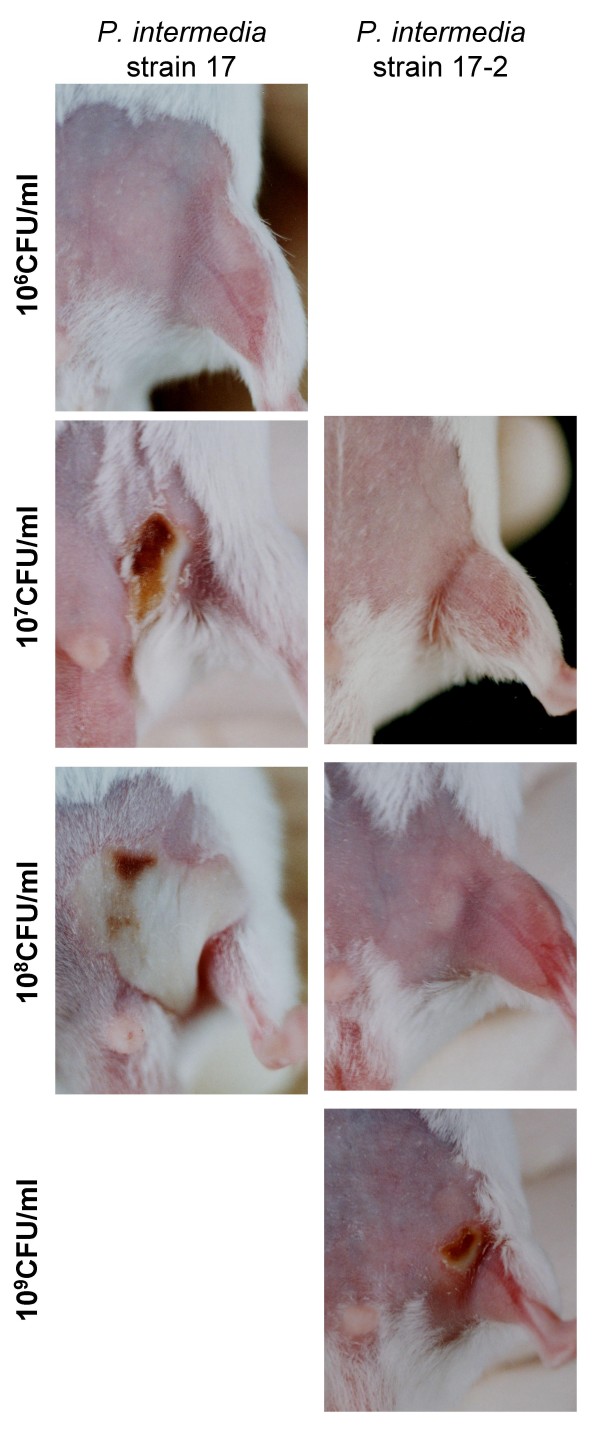
**Abscess induction in mice**. Abscess formation was induced when 0.5 ml of bacterial cell suspension (3 × 10^7 ^CFU/ml) of strain 17 was injected into the inguinal area of a mouse (left panels). In contrast, the subcutaneous injection of strain 17-2 (0.5 ml at a concentration of 10^7 ^and 10^8 ^CFU/ml) failed to induce an abscess in mice (right panels). Relatively small abscesses were induced when a higher cell concentration of strain 17-2 (10^9 ^CFU/ml) was injected (right bottom panel). The data are from one of three independent experiments.

To examine the influence of the biofilm phenotype on pathogenicity of *P. intermedia*, the abilities of strains 17 and 17-2 to induce abscesses in mice were compared. An injection of 500 μl of strain 17 at a concentration of 10^7 ^CFU/ml induced abscesses in mice (Fig. 8, left panel). In contrast, injection of a similar amount of strain 17-2 at the same growth phase did not induce abscesses in mice. A much higher cell concentration (10^9 ^CFU/ml) of strain 17-2 was required to induce abscesses in mice (Fig 8, right panel). However, an injection of a similar concentration of strain 17 was lethal for mice (data not shown).

### Internalization of bacterial cells by human PMNLs

**Figure 9:**
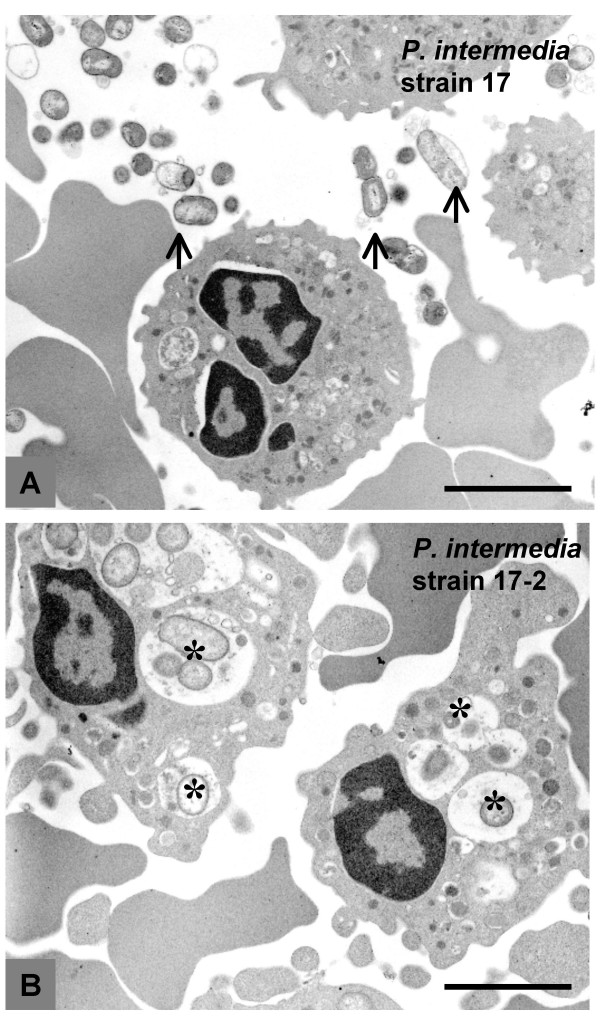
**Resistance of viscous material-producing strain 17 against the phagocytic activity of human neutrophils**. Strain 17 cells were not internalized by neutrophils though many of these cells were bound to the cell surface of neutrophils (A, arrows). In contrast, viscous material non-producing strain 17-2 cells were internalized and the ingested bacteria appear to be enclosed within cytoplasmic vacuoles (B, asterisks). Bars = 2.8 μm.

In the phagocytosis experiments, strain 17 cells were rarely internalized, though many of these cells were bound to the cell surface of PMNLs (Fig. 9A). In contrast, strain 17-2 cells were readily internalized by PMNLs after 90 min incubation. Many of these bacteria were found in cytoplasmic vacuoles (Fig. 9B).

### Gene expression profiles of strain 17 in biofilm *in vitro*

We next attempted to compare gene expression patterns of strain 17 between in biofilm and in planktonic conditions *in vitro*. Total RNA was isolated from 12 h cultures of strain 17 on solid culture media as its biofilm-forming cells and liquid cultures as planktonic cells, respectively. When the microarray expression data were compared, a total of 25 genes were up-regulated by at least four-fold with statistic significance (p < 0.05) in solid culture condition (Table 4). The expression of several genes which including those for a levanase (PINA0149), an extracytoplasmic function (ECF)-subfamily sigma factor (putative σ^E^: PINA0299), a putative lipoprotein (PINA1510), and a putative polysialic acid transport protein (KpsD, PINA1911) were protruded. Among hypothetical proteins, PINA1526 (putative CpxP) showed extremely high levels of transcription.

**Table 4:** Genes showing at least four-fold higher expression levels in biofilm-forming *Prevotella intermedia *strain 17 than those of strain 17 in planktonic condition

Gene	Fold change	Annotation
PIN0036	4.67	Hypothetical protein
PINA0141	6.78	Lipoprotein, putative
PINA0149	12.45	Levanase, ScrL
PINA0150	6.76	Levanase, SacC
PINA0151	4.71	Glucose-galactose transporter, putative
PINA0152	4.80	Fructokinase
PINA0194	4.02	Outer membrane protein
PINA0298	10.42	Hypothetical protein
PINA0299	9.16	ECF-subfamily sigma factor (σ^E^, putative)
PINA0300	5.62	Hypothetical protein
PINA0612	7.21	Hypothetical protein
PINA0990	4.24	Fibronectin type III domain protein
PINA1157	10.88	Hypothetical protein
PINA1452	4.24	Ribose-5-phosphate isomerase B
PINA1494	9.65	Hemin receptor, putative
PINA1510	18.41	Lipoprotein, putative
PINA1525	16.93	Hypothetical protein
PINA1526	28.60	Hypothetical protein with LTXXQ motif (CpxP, putative)
PINA1665	5.84	Hypothetical protein
PINA1807	7.24	Cell surface protein
PINA1833	4.16	AraC family transcriptional regulator
PINA1911	10.24	Polysialic acid transport protein, KpsD
PINA1931	4.06	Alkyl hydroperoxide reductase, subunit C, AhpC
PINA2066	8.94	Dps protein
PINA2119	4.99	Agmatinase, SpeC

## Discussion

It is well known that bacteria assuming biofilm-forming capacity have enormous advantages in establishing persistent infections even though they appear to be innocuous in their planktonic state [18-20]. Exopolysaccharide (EPS) is one of the main constituents of the biofilm extracellular matrix [21], and recent investigations have revealed that each biofilm-forming bacterium produces distinctive EPS components *e.g*. alginate and/or Psl found in *Pseudomonas aeruginosa *[22], acidic polysaccharide of *Burkholderia cepacia *[23], collanic acid, poly-β-1,6-GlcNAc (PGA) or cellulose found in *Escherichia coli *[24-27], cellulose of *Salmonella *[24,28], amorphous EPS containing N-acetylglucosamine (GlcNAc), _D_-mannose, 6-deoxy-_D_-galactose and _D_-galactose of *Vibrio cholerae *[29], polysaccharide intercellular adhesin (PIA) of *Staphylococcus *[30], and glucose and mannose rich components found in *Bacillus subtilis *biofilm [31]. In this study we found that *P. intermedia *strain 17 produced a large amount of EPS, with mannose constituting more than 80% of the polysaccharides. Among oral bacteria, the production of mannose-rich polysaccharide by *Capnocytophaga ochracea *has been reported [32]. This EPS provides a protection from attack by the human innate immune system [33]. We have also reported that a clinical isolate of *Prevotella nigrescens *can produce a copious amount of mannose-rich EPS [16].

In this study, biofilm-forming *P. intermedia *strain 17 showed stronger ability to induce abscesses in mice than that of strain 17-2, which was a naturally occurring variant of strain 17 that did not produce surface-associated fibrous material and therefore not capable of forming a biofilm. It is evidently shown that the slime/EPS production is critical for bacteria to exhibit the resistance to the neutrophil phagocytosis [33-36], though some EPS are not essential to bacterial adherence to host cells or for systemic virulence [37,38]. Jesaitis *et al*. [39] demonstrated that human neutrophils that settled on *P. aeruginosa *biofilms became phagocytically engorged, partially degranulated, and engulfed planktonic bacteria released from the biofilms. Deighton *et al*. [40] compared the virulence of slime-positive *Staphylococcus epidermidis *with that of slime-negative strain in a mouse model of subcutaneous infection and showed that biofilm-positive strains produced significantly more abscesses that persisted longer than biofilm-negative strains. TEM observation in our previous [16] and this study showed that *P. nigrescens *as well as *P. intermedia *with mannose-rich EPS appeared to be recognized by human leukocytes but not internalized. Leid *et al*. [41] have shown that human leukocytes can easily penetrate *Staphylococcus aureus *biofilms but fail to phagocytose the bacteria. Though we have to carefully investigate the possibility that multiple mutations exist in strain 17-2 and lead to the observed incapability to induce abscesses in mice, it is conceivable that biofilm bacteria being held together by EPS as in this case with strain 17 might present a huge physical challenge for phagocytosing neutrophils. In our previous study [16], we observed the restoration of the induction of abscess formation in mice when the purified EPS from the biofilm-forming strain of *P. nigrescens *was added to the cultures of a biofilm-non-forming mutant and injected into mice. As a consequence of these neutrophils being frustrated by their inability to phagocytose this bacterial mass, this might trigger the unregulated release of bactericidal compounds that could cause tissue injury as shown in the inflammatory pathway associated with lung injury [42,43] or chronic wounds [44]. The cellular components from neutrophils themselves are known to exert a stimulatory effect on the developing *P. aeruginosa *biofilm when the host fails to eradicate the infection [45].

Bacterial biofilm formation is likely to involve a cascade of gene expression events associating with a crossover of many sensing systems directed against environmental changes [46]. When we compare the microarray expression data obtained from strain 17 as bacterial cells were producing EPS to those of strain 17-2 as EPS non-producing variant, stress inducible heat shock proteins were up-regulated in strain 17 at a gene transcriptional level. We can not particularize functions of these genes in biofilm formation of *P. intermedia *since a genetic transfer system for having gene-targeted mutants of this organism yet remains to be developed [47,48]. However, recent studies evidently showed a tight relation between stress responses and biofilm formation [46,49-55], though stress response genes are not prominently up-regulated in some experimental biofilm formation [56]. We found in our earlier study that exposing biofilm-positive *P. intermedia *to environmental stress such as animal passages of the organism resulted in the up-regulations of HSPs at a protein level with increased production of cell surface-associated meshwork-like structures. By contrast, animal passages induced neither the production of viscous materials nor the up-regulation of HSPs in strain 17-2 (unpublished data).

When we compared the gene expression profiles of strain 17 cells plated on BAPs to those of planktonic cells in enriched-TSB, transcriptional levels of several genes including those for a levanase (ScrL: PINA0149), putative σ^E ^(PINA0299) and a polysialic acid transport protein (KpsD: PINA1911) were dramatically up-regulated on cells from the solid culture media. The highest transcriptional level was observed on a hypothetical protein (PINA1526) with LTXXQ motif which is found in a number of bacterial proteins bearing similarity to the protein CpxP [57]. PINA0299 (putative σ^E^) is homologous to the gene for AlgU which affects the conversion to mucoidy and alginate production in *P. aeruginosa *[58]. The AlgU (σ^E^)-dependent promoter of RpoH, well known positive regulator of heat shock genes, is known to be activated in mucoid type *P. aeruginosa *[58]. Although plating of planktonic cells at an exponential phase itself is known to immediately induce the expression of heat shock regulons in *E. coli *[59], we now hypothesize that, like AlgU (σ^E^) in *P. aeruginosa *[58], *P. intermedia *strain 17 cells keep their stress response via one of ECF sigma factors activated; thus rendering this organism to maintain EPS production at high levels in different growth conditions. However, so far we studied, gene clusters responsible for mannose-rich EPS still remain to be elucidated. To address the question of whether the gene expression phenomena observed in this study represent gene expression events behind the EPS production in *P. intermedia *biofilm, operon/genes for EPS synthesis regulated by stress-responsive systems of this organism must be explored in future studies.

## Conclusion

The data obtained in this study suggest that the *Prevotella *biofilms mainly composed of mannose-rich polysaccharides contribute to their resistance to host innate defence responses resulting in the development of chronic infections *in vivo*, and may also suggest that stress responsive systems of this organism might be behind its biofilm formation. To figure out a biofilm formation-gene expression relay system in *P. intermedia *requires the development of a suitable molecular tool that is capable of introducing specific targeted mutagenesis on genes highlighted in this study.

## Methods

### Bacterial strain and cultures

A viscous material producing clinical isolate of *P. intermedia*, which was isolated from a periodontitis lesion and designated as strain 17 [12], was used in this study. A total of 10 frozen culture stocks of isolated strain 17 were used in this study. Stock cultures of strain 17 in each vial were grown on trypticase soy blood agar plates (BAP) supplemented with 0.5% yeast extract (Difco Laboratories, Detroit, MI), hemin (5 mg/l), L-cystine (400 mg/l) and vitamin K_1 _(10 mg/l) or grown in the enriched-TSB: trypticase soy broth (TSB; BBL Microbiology Systems, Cockeysville, ND) supplemented with 0.5% yeast extract, hemin (5 mg/l), L-cystine (400 mg/l) and vitamin K_1 _(10 mg/l). Bacterial cultures were grown anaerobically in an anaerobic chamber (ANX-3, Hirasawa, Tokyo, Japan) at 37°C in a 5% CO_2_, 10% H_2_, 85% N_2 _atmosphere.

### Biofilm phenotype on strain 17 stock cultures

The ability to produce viscous materials in culture media and form meshwork-like structures on cell surfaces were used as criteria for distinguishing between "biofilm-forming" and "biofilm-non-forming" as described previously [16]. We first examined whether strain 17 met the criteria for being a biofilm-forming bacterium, since more than a decade has passed when we first described the unique phenotypic characteristic of strain 17 for its ability to produce viscous material [12]. Ten culture stocks were plated on BAP respectively and grown for 48 h anaerobically. Single colony from each culture stock was transferred to enriched-TSB and grown for 24 h as the seed culture. One hundred and fifty μl of this seed culture was transferred to enriched-TSB (15 ml) and grown for 48 h. The spent culture medium (550 μl) was put into a rotor, and the viscosity was measured as shearing stress between a rotor and a rotor shaft at 50 rpm, 20°C using a rotary viscometer (Toki-sangyo, Tokyo, Japan).

To examine cell surface structures, scanning electron microscopy (SEM) was performed. Bacteria grown on BAP for 48 h were collected on a piece of filter paper (Glass fiber GA55, Toyo Roshi, Tochigi, Japan), fixed with 2% glutaraldehyde in 0.1 M phosphate buffer for 2 h and 1% OsO_4 _in 0.1 M phosphate buffer for 1 h at 4°C, and dehydrated through an ethanol series and 2-methyl-2-propanol followed by platinum ion coating (E-1030, Hitachi, Tokyo, Japan). Specimens were examined with a scanning electron microscope (S-4800, Hitachi) at an accelerating voltage of 3 kV.

During the evaluation for the ability of our stock strain 17 cultures to form biofilms, one of the 10 stocks that we tested was a naturally-occurring variant that lacked the ability to form biofilms. A stock strain, designated as strain 17-2, produced neither viscous materials in culture medium nor cell surface-associated meshwork-like structures was obtained and considered as a biofilm-negative variant. These phenotypes were stably maintained in this variant strain despite repetitive passages *in vitro *or in animals (data not shown). The enzymatic activities of strains 17 and 17-2 were examined using the API ZYM system (bioMerieux, Marcy l'Etoile, France) and there was no significant difference regarding the production of enzymes (data not shown).

### Biofilm formation assay

The ability to form biofilm was investigated for strains 17 and 17-2 using crystal violet microtiter plate assay. Briefly, the seed cultures of both strains were prepared as described above and diluted to an OD of 0.1 at 620 nm in the same medium. Next, 150 μl diluted culture was transferred to each of eight sterile polystyrene microtiter plate wells (IWAKI, Tokyo, Japan) per strain. Sterile enriched-TSB was used as a control. The plates were prepared in duplicate and incubated at 37°C for 24 and 48 h, respectively. Biofilm formation was quantified according to Mohamed *et al*. [60]. This assay was repeated three times. A statistical analysis was performed using Student's *t*-test.

### Sugar composition of viscous materials from strain 17 cultures

The exopolysaccharide was prepared from culture supernatants by the method of Campbell *et al*. [61]. Briefly, *P. intermedia *strain 17 was grown at 37°C in enriched-TSB for 24 h. Supernatants were separated by centrifuging the liquid culture at 12,000 × *g *for 30 min, and sodium acetate was added to a final concentration of 5%. The mixture was stirred for 30 min at 22°C and the exopolysaccharide was isolated by ethanol precipitation from the reaction mixture. The ethanol-precipitated material was collected by centrifugation (18,200 × *g *for 15 min at 22°C), resolved in 5% sodium acetate, and treated with chloroform: 1-butanol (1: 5 by volume). Water-soluble and chloroform-butanol layer were separated by centrifugation, an equal amount of ethanol was added to the water-soluble layer (this procedure was repeated twice), and the ethanol-precipitated material was freeze-dried and stored at -80°C until use. Contaminated lipopolysaccharides (LPS) were removed from preparations according to the method of Adam *et al*. [62]. The freeze-dried material was dissolved in distilled water (0.5 mg/ml), and Triton X-114 (MP Biomedicals, Eschwege, Germany) stock solution (lower detergent rich phase) was added to a final concentration of 1% (v/v). After cooling on ice for 30 min, the solution was stirred at 4°C for 30 min and incubated at 37°C until the separation into two layers was complete. The upper aqueous phase was recovered by centrifugation for 30 min (1,000 × *g*, 30°C). This Triton X-114 treatment was performed twice. The upper aqueous phase was extracted three times with 3 vol CHCl_3_/CH_3_OH (2: 1 by volume) to remove detergent. The aqueous phase was concentrated under reduced pressure and freeze-dried. The contaminated-LPS level was measured by Limulus Amebocyte Lysate test according to the manufacturer's protocol (Seikagaku-kogyo, Tokyo, Japan).

The sugar composition of the purified viscous material were determined by means of HPLC for neutral and amino sugars and colorimetry for uronic acid. Briefly, neutral monosaccharides were released from purified exopolysaccharide (5 mg) by hydrolysis in a sealed tube with 2 N trifluoroacetic acid (200 μl) at 100°C for 6 h. The hydrolysate was concentrated *in vacuo *and dissolved in 500 ml of distilled water. The sugars were identified by HPLC (LC-9A, Shimadzu, Kyoto, Japan) with a TSK-gel sugar AXG column (15 cm × 4.6 mm) (Tosoh, Tokyo, Japan) using 0.5 M potassium tetraborate buffer (pH 8.7) as a carrier at a flow rate of 0.4 ml/min and a column temperature of 70°C. Amino sugars were released from purified exopolysaccharide (5 mg) by hydrolysis in a sealed tube with 4 N HCl (200 μl) at 100°C for 6 h. The hydrolysates were analyzed by HPLC (LC-9A, Shimadzu).

### Transmission electron microscopy of purified viscous materials

For negative staining, the ethanol precipitated viscous material was dissolved in distilled water (1 mg/ml). Fifteen microliters of the sample was deposited onto a formvar-coated and carbon-stabilized copper grid. After 1 min, excess fluid was removed with filter paper strips, stained with 2% uranyl acetate for 1 min, and examined in a transmission electron microscope (TEM) (H7100, Hitachi, Tokyo, Japan) at 100 kV.

### Microarray construction

To create a whole-genome microarray for *P. intermedia *strain 17, 30 perfect-matched and 30 miss-matched 24-mer probes were designed for all putative open reading frames (ORFs) (2,816 ORFs/array) from a whole genome sequence of *P. intermedia *strain 17, which is available from the Institute for Genomic Research data base (TIGR) using a Maskless Array Synthesizer (NimbleGen Systems Inc., Madison, WI, USA).

### RNA isolation

To determine an appropriate time point for total RNA isolation from the cultures of strains 17 and 17-2, morphological changes of cell surface structures associating with growth were examined by SEM. Single colony of Strains 17 and 17-2 grown on BAP for 24 h were inoculated into enriched-TSB and grown for 24 h as the seed culture. Five ml of this seed culture was used to inoculate 500 ml of enriched-TSB. The growth of the culture was monitored by measuring the absorbance at the wavelength of 600 nm. The morphology of cultured cells at a different stage of growth was examined by SEM as described above. RNA isolation was performed at a time point (12 h) when the surface-associated meshwork-like structure had begun to form. Total RNA samples were extracted from 12 h cultures of strains 17 and 17-2 using RNeasy Midi Kit (QIAGEN, Tokyo, Japan) according to the manufacturer's protocol. Samples were quantified and checked for purity using an Agilent 2100 bioanalyzer (Agilent, Hachioji, Japan). Total RNA (12 μg) was primed with random primer (Invitrogen, Tokyo, Japan), and cDNA was synthesized with reverse transcriptase (Superscript II, Invitrogen). The resulting cDNA was fragmented with DNase I (Promega, Madison, WI, USA) and labeled with biotin using terminal deoxynucleotidyl transferase (Promega). Biotin-labeled samples were hybridized onto the strain 17 microarray at 45°C for 16-20 h using NimbleGen's Hybriwheel Hybridization chambers (NimbleGen Systems Inc.).

To compare gene expression profiles of strain 17 in solid and liquid culture conditions, seed cultures of strain 17 were newly prepared as described above. Five ml of this seed culture was transferred to enriched-TSB (500 ml) and 200 μl of the seed cultures was transferred to each of 50 BAPs. Both cultures were incubated for 12 h anaerobically. Total RNA was isolated from the liquid cultures as described above. Two hundred μl of PBS was added to BAPs to harvest growing cells using cell scrapers (IWAKI). Cell suspensions were washed twice with PBS and total RNA was isolated as described above.

### Microarray image acquisitions and data analyses

Hybridized-microarray slides containing technical duplicates were imaged with a high resolution array scanner (GenePix 4000B Microarray Scanner, Molecular Devices Corp., Sunnyvale, CA, USA) and the fluorescent signal intensities from each spot were quantified using NimbleScan Software (NimbleGen Systems Inc.). Normalization was performed among four microarray hybridization data sets by means of Robust Multi-chip analysis algorithm [63] and statistical analyses were performed using *t*-test and Bonferroni adjustment in the Roche-NimbleGen Microarray soft wears (Roche Diagnostics, Tokyo, Japan). When the individual probes met the criteria that the average signals from the culture of biofilm-positive strain versus the average signals from biofilm-negative strain were different by at least twofold with statistic significance, probes selected were used to find up-regulated regions.

Pertinent information on raw data containing experimental designs and hybridization results for specific oligonucleotide sets is available in CIBEX database [17].

### Quantitative real-time RT-PCR

To confirm the up-regulation of several genes in strain 17 recorded by the microarray, a real-time RT-PCR strategy was employed. Twelve hours cultures of strains 17 and 17-2 were prepared again and total RNA was isolated as described above. Real-time RT-PCR was performed according to the one-step RT-PCR protocol of iScript™ One-Step RT-PCR Kit with SYBR^® ^Green (BIO-RAD Laboratories, Tokyo, Japan). Briefly, 50 ng of total RNA, 200 nM of forward and reverse primers for a target gene, and 25 μl of SYBR^® ^Green RT-PCR Reaction Mix (BIO-RAD Laboratories) were added into a PCR tube containing one μl of iScript Reverse Transcriptase for One-Step RT-PCR. The PCR preparation was brought to a final volume of 50 μl with nuclease-free water (BIO-RAD Laboratories). As an internal control, RT-PCR for 16S rRNA was performed at 50°C for 10 min, 95°C for 5 min, followed by 35 cycles at 95°C for 10 sec and 64°C for 30 sec followed by melt curve analysis. Primers for 16S rRNA and several genes showing up-regulation in microarray assays were as follows: 16S rRNA: forward 5'-AGAGTTTGATCCTGGCTCAG-3', reverse 5'-AAAGGAGGTGATCCAGCC-3'; PIN 0281: forward 5'-TGAACGTAAGCCGCAGCTAC-3', reverse 5'-TTGTTCTTGGCGCAAAGCAG-3'; PINA1058: forward 5'-TGTGAACCCCGACGAAGTGG-3', reverse 5'-GTGCCTGCTGACCAGCATCT-3'; PINA1756: forward 5'-AATACAGCCTTCGAGGGTTT-3', reverse 5'-TTCGGTCAAGACAGTAGGGA-3'; PINA1797: forward 5'-TGAAGATTTGCGCTGTCAAG-3', reverse 5'-TAGCAGGAGTTTCTTCAGGT-3'; PINA1798: forward 5'-AGCGGAGCAGAAAGTAGGTG-3', reverse 5'-CAACAGCAAGAACGTCGCTT-3'; PINA2006: forward 5'-CTTGGAACAACGGGTACAGG-3', reverse 5-AAATCTCGCTTTGCGTCAGT-3'. Annealing temperatures were optimized for each primer pair by the use of melting curve analysis in which the melting curve starts at 55°C and ends at 90°C with temperature increment of 0.2°C and a hold time of 2 sec. The optimized annealing temperature for each target gene was 64.5°C for PIN 0281, 62.0°C for PINA1058, 64.5°C for PINA1756, 65.0°C for PINA1797, 58.7°C for PINA1798 and 57.6°C for PINA2006, respectively. The threshold cycle (C_T_) values were obtained for the reactions reflecting the quantity of the template in the sample. ΔC_T _for each gene was calculated by subtracting the calibrator gene 16S rRNA C_T _value from each of the target values represented the relative quantity of the target mRNA normalized to the level of the internal standard 16S rRNA mRNA level. The target mRNA levels in strains 17 and 17-2 were defined and compared.

To observe how the expression levels of these genes fluctuate through the culture period, single colony of strains 17 and 17-2 grown on BAP for 24 h were inoculated into enriched-TSB and grown for 24 h as the seed culture. One hundred and fifty μl of this seed culture was used to inoculate 15 ml of enriched-TSB. Total RNA samples were extracted from 6, 12, 18, 24 and 30 h cultures of strains 17 and 17-2 using RNeasy Midi Kit (QIAGEN) and applied to the real-time RT-PCR as described above. Changes of the target mRNA levels through the culture period were recorded by the strain.

### Animal studies

The virulence of biofilm-forming strain 17 was compared with that of biofilm-non-forming variant strain 17-2 regarding abscess formation in mice. Bacterial strains were cultured in enriched-TSB for 24 h for strain 17-2 and 36 h for strain 17, respectively (early stationary phase; see Fig. 5). Five hundred μl of bacterial suspensions (10^6 ^to 10^10 ^CFU/ml) was injected subcutaneously into the inguen of each BALB/c mouse (male, 4 weeks; 3 mice per strain). Changes of abscess lesions were recorded photographically using a camera (Nikon FIII, Nikon, Japan) set at a fixed magnification for five consecutive days.

### Phagocytosis assay

To compare anti-phagocytic activity of strain 17 with that of strain 17-2, bacterial cells were co-cultured with polymorphonuclear leukocytes (PMNL) obtained from healthy human volunteers (n = 3; age 20–23 years) in accordance with institutional approved procedures. PMNLs were purified by a PolymorphprepTM (Axis-Shield PoC AS, Oslo, Norway) gradient and cultured in RPMI 1640 medium (Gibco BRL, Grand Island, NY) supplemented with 10% heat-inactivated fetal calf serum (FCS) (MultiSer, Trace Scientific Ltd, Melbourne, Australia) and 2 mM L-glutamine at 37°C in 5% CO_2_. Bacterial cell suspensions (1.5 × 10^6 ^CFU/ml) were prepared from strains 17 and 17-2 cultures as described in the animal studies. Three hundred μl of PMNLs (10^6 ^cells/ml) was dispensed into the wells of 24-well tissue culture plates (Becton Dickinson, Franklin Lakes, NJ). To these wells, 100 μl of bacterial suspension of different tested strains was added. After incubation for 60–90 min at 37°C, PMNLs co-cultured with bacterial cells were centrifuged at 8,000 × *g *at 4°C for 5 min and processed for transmission electron microscopy to determine the internalization of tested strains by PMNLs. Cell pellets were fixed with 2% glutaraldehyde in 0.1 M phosphate buffer for 2 h at 4°C, post-fixed with 1% OsO_4 _in 0.1 M phosphate buffer for 1 h at 4°C, and dehydrated through an ethanol series. Samples were embedded into Epon resin and ultrathin sections were prepared by a ultramicrotome (Ultracut, Leica, Tokyo, Japan). Ultrathin sections were placed on a copper grid, stained with uranyl acetate and lead citrate, and observed in a TEM (H7100, Hitachi).

## Authors' contributions

TY, TF and CM carried out the phenotype characterization and microarray analysis, and drafted the manuscript. KY and CS performed RT-PCR. NM and HN screened a culture collection of strain 17 for the ability to produce viscous material. TN participated in the analysis of microarray data. CBW, KPL, and HF participated in the design of this study and drafted the manuscript.
